# Clinical and Magnetic Resonance Imaging Findings of the Temporomandibular Joint in Patients With Rheumatoid Arthritis

**DOI:** 10.4021/jocmr1084w

**Published:** 2012-09-12

**Authors:** Ozcan Hiz, Levent Ediz, Yasemin Ozkan, Aydin Bora

**Affiliations:** a Yuzuncu Yil University Medical Faculty, Physical Medicine Rehabilitation and Rheumatology Department, Van, Turkey; b Yuzuncu Yil University Medical Faculty, Radiology Department, Van, Turkey

**Keywords:** Rheumatoid arthritis, Temporomandibular joint, Magnetic resonance imaging, Jaw clenching force, Mouth opening

## Abstract

**Background:**

The aim of this study is to evaluate temporomandibular joint (TMJ) involvement in patients with rheumatoid arthritis by magnetic resonance imaging (MRI), jaw clenching force, mouth opening, and Fonseca’s questionnaire, and to establish the relationship between these findings and clinical, radiologic, and laboratory activity parameters that are unique to rheumatoid arthritis.

**Methods:**

Included in the study were 30 RA patients and 30 healthy volunteers. Jaw clenching force of the entire cases was measured with Istanbul Bite Force Recorder (kg) and the mouth opening was measured with a ruler (cm). Additionally, hand grip forces of patients with rheumatoid arthritis were measured with hand dynamometer (kg). Hand and feet graphs and TMJ MRIs of patients were obtained. MRI findings were classified as normal, mild, medium, and severe. DAS28 and sharp scores of patients were estimated. Sedimentation rate (ESR), C-Reactive protein (CRP) and rheumatoid factor (RF) were checked in the patient group and Fonseca’s questionnaires were filled in.

**Results:**

A significant difference was not observed between age, gender, and level of education of the groups. Jaw clenching force and mouth opening were established as significantly low in RA group compared to the control group (P < 0.001). A significant correlation was found between jaw clenching force, hand grip force, mouth opening, questionnaire, and MRI findings with the disease duration sharp score, DAS28, and hand grip force of the RA group (P < 0.05). However, a significant correlation was not established with ESR, CRP, and RF (P < 0.001).

**Conclusions:**

Jaw clenching force, mouth opening, and Fonseca’s questionnaire can be used as parameters pointing to TMJ involvement in patients with RA. Yet, further studies in which TMJ involvement is followed up since the onset of the disease are of necessity.

## Introduction

Rheumatoid arthritis is a systemic chronic inflammatory disease of which the aetiology is not fully known and that affects joints dominantly. Temporomandibular joint involvement in patients with RA is a well-known condition. Due to the selection of patient population and methods used for diagnosis, its prevalence greatly differs in each study. In order to demonstrate TMJ involvement, such radiologic methods as direct radiography, ultrasonography, tomography, and MRI have been utilized [1-13]. In previously conducted studies, the relationship between general disease activity and temporomandibular joint involvement in RA patients have been established, and TMJ involvement was reported to be between 2-88% [1-10]. In these studies, correlations were reported to exist between age, duration of the disease, number of swollen joints, RF, ESR, CRP, thrombocyte count, and plasma tumor necrosis factor-alpha levels with temporomandibular joint involvement [5, 8, 14-16]. Based on these studies, TMJ involvement is more prevalent in severe and late-stage RA patients.

In patients with rheumatoid arthritis, decrease in hand grip force and functional disorders appear due to proliferation and inflammation of synovial tissue in wrists and hand joints [17]. We moved from the idea that, similar to the decrease in hand grip force, a decrease in jaw tightening force could also be possible in RA patients with TMJ involvement. However, we have yet to come across a study that compares the jaw tightening force in patients with RA with healthy control groups and other clinical findings of RA. The primary objective of this study is to examine the effect of TMJ involvement established in patients with RA on jaw tightening force and mouth opening, and secondarily, to reveal the correlation of MRI, jaw tightening force, and mouth opening with disease duration, DAS28, hand grip force, sharp score, CRP, sedimentation, and RF.

## Materials and Methods

### Cases

In this study, 117 RA patients in the age group of 18 - 65 that meet the criteria of American Rheumatism Association were evaluated. Examinations of the entire cases were conducted by a specialist at the Medicine Faculty Physical Medicine and Rehabilitation clinic. Included in the study were patients who have been diagnosed with RA at least six months before and received DMARD + steroidal/nonsteroidal treatment. Patients that previously have had jaw-related traumas, with teeth and gum diseases to the extent that it affects jaw force, total or partial upper or lower dentures, bruxism history, facial nerve paralysis, cerebrovascular event, trigeminal neuralgia, and polyneuropathy history, active psychiatric disease history, diseases with juvenile onset, and that underwent TMJ injection in the last six months were excluded from the study. In addition, Turkish-illiterate patients were also excluded from the study. At the beginning of the study, all the patients were asked to tell their painful joints. Following the implementation of inclusion and exclusion criteria of the study, 30 patients and a same number of healthy volunteers underwent tests. This controlled clinical and radiological study was approved by the Medicine Faculty Ethics Committee of the University, and the entire cases were verbally informed about the study and their consents were obtained.

### Clinical, radiological, and laboratory assessments

Maximum mouth opening of the patient and control groups were measured with a ruler in centimeter. Jaw tightening force of the patient and control groups were measured with Istanbul Bite Force Recorder from right and left. In order to establish the degree of TMJ dysfunction, Fonseca’s questionnaire was filled in. DAS28 and Sharp scores of the patient group were estimated. MRI of each temporomandibular joint was carried out, and in addition, erythrocyte sedimentation rates, C-Reactive protein, and rheumatoid factor levels of patients were established.

### TMJ dysfunction questionnaire (Fonseca’s questionnaire)

In order to evaluate TMJ function, a questionnaire that was developed by Fonseca in 1992 to establish the symptoms and severity of temporomandibular joint diseases were filled in for the RA group [18]. This questionnaire is comprised of 10 questions, and each question is replied as no (0 points), sometimes (5 points), and yes (10 points). Total score obtained from the said questionnaire demonstrates normal TMJ function if between 0 - 15, mild TMJ dysfunction if between 20 - 40, and medium TMJ dysfunction if between 45 - 65, and severe TMJ dysfunction if between 70 - 100.

### Jaw tightening force measurement

Maximal bite force (MBF) of patients with rheumatoid arthritis and healthy individuals included in the study were measured in kilograms with Istanbul Bite Force Recorder^TM^ from the incisal region. Istanbul Bite Force Recorder has been designed as a portable device used as a strain gage in order to obtain bite force recordings [19]. The device has been put in the mouth so that the sensors were placed between central incisors while teeth were in interincisal position. Sterile plastic caps were utilized for each patient and the device was cleaned with disinfectant solution. The measurements were carried out twice with 10 days apart, and averages were obtained. Additionally, hand grip forces of the patient group were measured by using a hand dynamometer.

### Radiological assessment (Magnetic resonance imaging, X-ray)

Tesla superconductive imaging system (Siemens Symphony, Erlangen, Germany) was used for visualization of the temporomandibular joint. MRI assessment was evaluated by a radiologist who was unaware of the clinical and laboratory information of the patients. Condylar structure, articular eminence morphology, translocation, disc position, and bone marrow changes were recorded in the MRI evaluation. MRI findings were divided into 4 categories similar to the study by Lin et al [10] in which they carried out imagings of TMJ by using tomography: Grade 0 (normal): normal condyle and joint structure, Grade 1 (mild): a mild irregularity in condyle, destruction, bone marrow changes, minimal joint space narrowing, Grade 2 (medium): significant erosion in condyle, destruction and joint space narrowing, Grade 3 (severe): complete destruction of condyle and joint space narrowing. Additionally, hand and foot x-rays were also obtained in order to estimate Sharp score of the patient group.

### Statistical analysis

Analysis of the data was conducted by using SPSS for Windows 13 software pack. Whether the distribution of continuous variables was near normal was investigated with Shapiro-Wilk test. Definitive statistics were demonstrated as average ± standard deviation or median (minimum-maximum) for continuous variables, and categorical variables were shown as the number of cases and (%). Significance of the difference in terms of averages between groups was investigated with Student’s t test, and significance of the difference in terms of median values was studied with Mann Whitney U test. Nominal variables were evaluated with Pearson’s Chi-Square test. The presence of a statistically significant correlation between continuous and enumerable variables was studied by Spearman’s Correlation test. The results were accepted as statistically significant for P < 0.05.

## Results

A difference was not present between the RA group and the control group in terms of age, gender, and level of education (P > 0.05). Demographic characteristics of both groups are given in Table 1. Of the cases, 20 had high CRP, 16 had high ESR, 24 had positive RF. Duration of the disease, DAS28 score, sharp score, right hand grip force, and left hand grip force values of the RA group are given in Table 2.

**Table 1:** Demographic Characteristics

Variables	Control Group	Patient Group	P-value
Age	42.0 ± 10.3	38.8 ± 11.6	0.264
Gender			0.754
Male	6 (20.0%)	7 (23.3%)	
Female	24 (80.0%)	23 (76.7%)	
Education Level			0.956
Literate	22 (73.3%)	20 (66.7%)	
Elementary	6 (20.0%)	7 (23.3%)	
Secondary	1 (3.3%)	2 (6.7%)	
High School	1 (3.3%)	1 (3.3%)	

**Table 2:** Disease Duration, DAS28, Sharp Score, and Hand Grip Forces in the Group With Rheumatoid Arthritis

Variables	n = 30
Disease duration (month)	63.3 ± 52.7
DAS28	4.9 ± 1.1
Sharp score	57.5 ± 30.6
Hand Grip Force-Right	10.0 ± 2.9
Hand Grip Force-Left	11.2 ± 2.6

Right and left jaw tightening force and mouth opening measurements of the RA group were established as low on a statistically significant level compared to the control group (P < 0.001) (Table 3).

**Table 3:** Jaw Tightening Force and Mouth Opening Comparison of Rheumatoid Arthritis and Control Groups

	Control Group	Case Group	P-value
Jaw Tightening Force-Right	28.5 (20 - 36)	12.5 (5 - 36)	< 0.001
Jaw Tightening Force-Left	28 (22 - 34)	12 (7 - 30)	< 0.001
Mouth Opening	5 (5 - 5)	3 (2.3 - 5)	< 0.001

Before any examinations and radiological tests were conducted in the patient group, 12 patients were established to report TMJ as painful joint. Notwithstanding, while TMJ disability was present in 29 patients in varying degrees based on Fonseca’s questionnaire, 1 patient was within normal limits. While 29 patients had TMJ involvement in varying degrees according to right TMJ MRI results, 1 patient was normal. And while 25 patients had TMJ involvement according to left TMJ MRI results, 4 patients were established as normal. Number and percentage of patients with TMJ involvement based on questionnaire and MRI results are given in Table 4.

**Table 4:** Distribution of Cases Based on the Degree of TMJ Involvement According to MRI and Fonseca’s Questionnaire in the RA Group

Involvement	MRI (Right TMJ)	MRI (Left TMJ)	Fonseca’s questionnaire
Normal	1 (3.3%)	4 (13.3%)	1 (3.3%)
Mild	11 (36.7%)	12 (40.0%)	3 (10.0%)
Moderate	13 (43.3%)	9 (30.0%)	18 (60.0%)
Severe	5 (16.7%)	5 (16.7%)	8 (26.7%)

MRI: Magnetic resonance imaging; TMJ: Temporomandibular Joint.

There was statistically significant correlation between entire parameters we checked in order to find out TMJ involvement (right and left jaw tightening force, mouth opening, Fonseca’s questionnaire, right and left TMJ MRI) (P < 0.001). Established correlations were negative between jaw tightening force and mouth opening with questionnaire and MRI, positive between right and left jaw tightening forces, and positive between questionnaire and MRI (Table 5).

**Table 5:** Correlations (CC) Between Temporomandibular Joint Parameters

Variables		Jaw Tightening Force (Right)	Mouth Opening	Fonseca’s Questionnaire	MRI (Right TMJ)	MRI (Left TMJ)
Jaw tightening force (Left)	CC	0.924	0.841	-0.749	-0.796	-0.833
	P	< 0.001	< 0.001	< 0.001	< 0.001	< 0.001
Jaw tightening force (Right)	CC		0.826	-0.686	-0.884	-0.707
	P		< 0.001	< 0.001	< 0.001	< 0.001
Mouth opening	CC			-0.569	-0.651	-0.610
	P			< 0.001	< 0.001	< 0.001
Fonseca’ Questionnaire	CC				0.679	0.856
	P				< 0.001	< 0.001
MRI (Right)	CC					0.709
	P					< 0.001

MRI: Magnetic resonance imaging; TMJ: Temporomandibular Joint.

When checked the correlations between parameters showing TMJ involvement and RA-specific disease activity and level, significant negative correlations were found between mouth opening and jaw tightening force with Sharp score, DAS28 and disease duration, and positive correlations were established between jaw tightening force and mouth opening. While a positive correlation was present between Fonseca’s questionnaire and MRI results with Sharp score, DAS28 and disease duration, there was a negative correlation with hand grip forces (a correlation was not present with right MRI and left hand grip force). A significant correlation was not established between mouth opening, jaw tightening force, Fonseca’s questionnaire, and MRI results with laboratory parameters (CRP, ESR, RF) (Table 6).

**Table 6:** Correlation Coefficient (CC) and the Level of Significance (P) Between Temporomandibular Joint Parameters With ESH, CRP, Hand Grip Force, Sharp Score, and DAS28

Variables		Sharp score	ESR	CRP	RF	HGF-Right	HGF-Left	DAS28	Disease duration
Hand grip force (left)	CC	-0.808	0.022	-0.006	-0.004	0.593	0.454	-0.924	-0.776
	P	< 0.001	0.908	0.973	0.982	< 0.001	0.012	< 0.001	< 0.001
Jaw tight force (right)	CC	-0.771	-0.116	-0.095	-0.032	0.552	0.562	-0.854	-0.735
	P	< 0.001	0.542	0.617	0.868	0.002	< 0.001	< 0.001	< 0.001
Mouth opening	CC	-0.668	0.067	0.071	-0.055	0.499	0.394	-0.75	-0.649
	P	< 0.001	0.725	0.711	0.774	0.005	0.031	< 0.001	< 0.001
Investigation	CC	0.820	-0.026	0.136	0.156	-0.473	-0.387	0.855	0.807
	P	< 0.001	0.893	0.474	0.411	0.008	0.035	< 0.001	< 0.001
MRI (Right)	CC	0.694	0.178	0.193	-0.005	-0.596	-0.197	0.785	0.706
	P	< 0.001	0.347	0.307	0.979	< 0.001	0.296	< 0.001	< 0.001
MRI (Left)	CC	0.755	-0.164	-0.142	-0.098	-0.472	-0.491	0.890	0.760
	P	< 0.001	0.386	0.453	0.608	0.008	0.006	< 0.001	< 0.001

ESH: erythrocyte sedimentation rate; CRP: C-Reactive protein; RF: Rheumatoid factor; MRI: Magnetic resonance imaging; ESG: Hand Grip Force.

## Discussion

Involvement of TMJ that is a synovial joint is an expected condition in rheumatic diseases. However, studies on TMJ involvement in RA patients are not too many in number. In our study, TMJ involvement was examined with MRI, mouth opening, jaw tightening force, and Fonseca’s questionnaire in patients with RA. A study compared jaw tightening force with radiological findings, disease activity, disease duration, and laboratory findings in patients with rheumatoid arthritis has not been found so far in the accessible electronic environment. We moved from the argument that a similar decrease in hand grip force developed in majority of patients due to inflammation in hand-wrist joints in RA patients could also occur in jaw tightening force. Based on our study results, TMJ involvement was established in about 975 of the patients with RA, and jaw tightening force and mouth opening in patients with involvement were found to be significantly low compared to the healthy group. As it is known, rheumatoid arthritis is a disease characterized by symmetrical joint involvement. In 26 of the RA patients in our study, TMJ involvement was established and 3 patients had asymmetrical joint involvement.

Different imaging techniques can be used in order to establish TMJ disorders. In previous studies, TMJ involvement was found by using x-ray [20], computerized tomography [10], and magnetic resonance imaging [21] methods. We used MRI in our study as it demonstrates joint inflammation better and has lower radioactivity level. We determined a scoring system from one to four in order to establish the level of TMJ involvement. Previous studies that utilized different radiological evaluation methods reported TMJ involvement in more than half of the patients with RA [1, 6, 10, 22-27]. In the study by Lin et al [10], TMJ complaints were established in 51.8% of the patients, and temporomandibular joint involvement in other patients was reported to be clinically insignificant. In our study, TMJ involvement was found in the majority (97%) of the patients according to MRI, Fonseca’s questionnaire, and clinical examination results of patients with RA. Only one patient did not have TMJ involvement based on MRI and questionnaire results. Although TMJ involvement determined by a physician was at higher rates in our study, only 12 (40%) of our patients described complaints related to temporomandibular joint. Even though some of our patients had severe joint involvement, they did not describe any complaint, however, those patients were established to have TMJ joint problems through detailed questioning, clinical examination, and MRI. Based on these results, TMJ involvement follows a clinically insignificant course in some patients as noted by previous authors [10, 28]. Among the reasons of this is that, due to unique structure of TMJ, retrodiscal tissue rich in blood vessels provides an easier resorption of exudate within the joint compared to other joints, thus, swelling and pain is decreased [10]. Another reason is that patients subjectify TMJ complaints and attempt to solve the problems by such methods as speaking less and avoiding hard-to-chew foods [29]. We believe, in addition to what previous authors noted, our patients may have been attaching more importance to other peripheral joint involvements that affect daily life activities and work lives bearing in mind the education and socio-economic levels of the region where the study was conducted and due to the fact that a majority of our patients were female (as mainly women are responsible for childcare and housekeeping chores). Besides, us physicians have less interest in TMJ in RA patients during the daily practice and this could cause ignoring TMJ involvement at the time of questioning the patient, meaning that, when the patients are asked what their complaints are, they might not report TMJ complaints possibly due to above-mentioned reasons, and they could probably only report any involvement when they are directly asked about TMJ. However, we did not determine by directly noting the name of joints for the purpose of not guide patients in our study. We conducted our investigations by asking from which joints they were having complaints.

Disease duration longer than five years in RA cases is believed to be a significant factor affecting TTMJ involvement [5, 10]. However, some studies reported that about 30% of patients had TMJ involvement within a year after the onset of RA [30]. Our study established the average disease duration as 63.3 ± 52.7. However, we determined the disease duration as the duration of diagnosis in our study. Therefore, the actual disease duration is probably much longer. Early diagnosis and appropriate treatment is of great importance in RA patients in order to prevent joint destruction [31, 32]. Thus, the patients included in our study were selected from among the patients that were under regular follow-up in our clinic and were receiving similar treatments (DMARD, and additionally, nonsteroidal/steroidal group drugs). Yet, the treatment doses were not standard. Additionally, due to the socio-cultural structure of our region, compliance with taking drugs is not quite good. All these reasons may have caused the appearance of TMJ involvement in our patient group more compared to other studies.

Such clinical findings as TMJ-related sensitivity, morning stiffness, clicks, crepitation, and maximal mouth opening have been used in previous studies [3, 20, 27]. In our study, we utilized maximal mouth opening and maximum jaw tightening force which has never been used in any other studies as clinical examination findings. We found in our study that maximal mouth opening was significantly lower compared to healthy control group as was the case in previous studies [3, 31]. Similarly, we established the maximal jaw tightening force as significantly lower in the RA group compared to the healthy control group (P < 0.001). In addition, we found a strong correlation between mouth opening and jaw tightening force (P < 0.001).

Laboratory indicators of inflammation are known to be in relation with joint damage. In previous studies, a correlation was found between CRP, ESR, RF, thrombocyte count, and tumor necrosis factor alpha levels with TMJ involvement [5, 10, 15, 16, 31, 33]. In a study by Lin et al, a correlation was established between RF and ESR with the severity of TMJ involvement, a correlation was not found with CRP [10]. In a study conducted in our country, a relation was reported between laboratory findings and TMJ involvement, however, condylar erosion was found in RF negative cases [27]. However, in our study, a significant correlation was not observed between CRP, ESR, and RF with TMJ involvement. We selected the cases to be included in our study from among regularly followed up patients since they were diagnosed. Yet, the average diagnosis duration of our patient group was established as about five years. Thus, TMJ involvement might have appeared during the time it took to diagnose. This might have contributes to the inability to establish a correlation between CRP, sedimentation, and RF with TMJ involvement. However, our study established a significant correlation between other parameters we checked to find the disease level (Sharp score and DAS28) and parameters we checked to establish TMJ involvement (P < 0.001) (Table 6). Our results demonstrate that TMJ involvement was correlated with radiological and clinical findings of RA, but not correlated with laboratory findings.

### Limitations of the study

In previous studies investigating TMJ involvement in patients with rheumatoid arthritis, patients with other rheumatic diseases (AS, psoriasis, systemic sclerosis, systemic lupus erythematosus, mixed connective tissue disease) [12, 22] and osteoarthritis [7, 32, 34, 35] were also included in the study as control group in addition to the healthy group. However, we only included healthy volunteers with no previous TMJ complaints as the control group. We were not able to conduct TMJ MRI in the healthy group. Thus, we could not compare RA and control group in radiological terms. Therefore, patients with osteoarthritis were probably present in the control group. But, in spite of this, we established a significant difference between the RA group and the control group in terms of jaw tightening force and mouth opening. Another restriction was that the Turkish validation and reliability of Fonseca’s questionnaire were not possible, and additionally, accuracy of the data might have proven to have problems due to the low level of education in our region. Besides, although the group we included in the study was selected from among the treatment group that was receiving DMARD and steroidal/nonsteroidal drugs, the treatment was not standard. Quite diverse drugs were in use in quite different doses. Due to this reason and the low number of patients meeting the inclusion criteria, we were not able to conduct a comparison on drugs.

Consequently, based on our study results, TMJ involvement is common in patients with rheumatoid arthritis. In order to prevent TMJ involvement, radiological and clinical diagnosis should be made as early as possible, and the most appropriate treatment should be commenced. To that end, examinations and imaging methods concerning TMJ should be utilized in addition to other peripheral joints in all patients with RA. While jaw tightening force, mouth opening, and Fonseca’s questionnaire are easily implemented, cheap, and beneficial methods to establish TMJ involvement in patients with RA, MRI helps to demonstrate the severity of TMJ involvement. However, further studies with longer follow-ups in which jaw tightening force and mouth opening is constantly measured since diagnosis and TMJ MRI is conducted are of necessity.

## References

[1] Ogus H (1975). Rheumatoid arthritis of the temporomandibular joint. Br J Oral Surg.

[2] Tegelberg A, Kopp S (1987). Clinical findings in the stomatognathic system for individuals with rheumatoid arthritis and osteoarthrosis. Acta Odontol Scand.

[3] Larheim TA, Smith HJ, Aspestrand F (1990). Rheumatic disease of the temporomandibular joint: MR imaging and tomographic manifestations. Radiology.

[4] Goupille P, Fouquet B, Cotty P, Goga D, Mateu J, Valat JP (1990). The temporomandibular joint in rheumatoid arthritis. Correlations between clinical and computed tomography features. J Rheumatol.

[5] Celiker R, Gokce-Kutsal Y, Eryilmaz M (1995). Temporomandibular joint involvement in rheumatoid arthritis. Relationship with disease activity. Scand J Rheumatol.

[6] Kallenberg A, Wenneberg B, Carlsson GE, Ahlmen M (1997). Reported symptoms from the masticatory system and general well-being in rheumatoid arthritis. J Oral Rehabil.

[7] Gynther GW, Holmlund AB, Reinholt FP, Lindblad S (1997). Temporomandibular joint involvement in generalized osteoarthritis and rheumatoid arthritis: a clinical, arthroscopic, histologic, and immunohistochemical study. Int J Oral Maxillofac Surg.

[8] Yoshida A, Higuchi Y, Kondo M, Tabata O, Ohishi M (1998). Range of motion of the temporomandibular joint in rheumatoid arthritis: relationship to the severity of disease. Cranio.

[9] Yamakawa M, Ansai T, Kasai S, Ohmaru T, Takeuchi H, Kawaguchi T, Takehara T (2002). Dentition status and temporomandibular joint disorders in patients with rheumatoid arthritis. Cranio.

[10] Lin YC, Hsu ML, Yang JS, Liang TH, Chou SL, Lin HY (2007). Temporomandibular joint disorders in patients with rheumatoid arthritis. J Chin Med Assoc.

[11] Akerman S, Jonsson K, Kopp S, Petersson A, Rohlin M (1991). Radiologic changes in temporomandibular, hand, and foot joints of patients with rheumatoid arthritis. Oral Surg Oral Med Oral Pathol.

[12] Helenius LM, Tervahartiala P, Helenius I, Al-Sukhun J, Kivisaari L, Suuronen R, Kautiainen H (2006). Clinical, radiographic and MRI findings of the temporomandibular joint in patients with different rheumatic diseases. Int J Oral Maxillofac Surg.

[13] Melchiorre D, Calderazzi A, Maddali Bongi, Cristofani R, Bazzichi L, Eligi C, Maresca M (2003). A comparison of ultrasonography and magnetic resonance imaging in the evaluation of temporomandibular joint involvement in rheumatoid arthritis and psoriatic arthritis. Rheumatology (Oxford).

[14] Goupille P, Fouquet B, Goga D, Cotty P, Valat JP (1993). The temporomandibular joint in rheumatoid arthritis: correlations between clinical and tomographic features. J Dent.

[15] Nordahl S, Alstergren P, Eliasson S, Kopp S (2001). Radiographic signs of bone destruction in the arthritic temporomandibular joint with special reference to markers of disease activity. A longitudinal study. Rheumatology (Oxford).

[16] Voog U, Alstergren P, Eliasson S, Leibur E, Kallikorm R, Kopp S (2003). Inflammatory mediators and radiographic changes in temporomandibular joints of patients with rheumatoid arthritis. Acta Odontol Scand.

[17] Veehof MM, Taal E, Heijnsdijk-Rouwenhorst LM, van de (2008). Efficacy of wrist working splints in patients with rheumatoid arthritis: a randomized controlled study. Arthritis Rheum.

[18] Nomura K, Vitti M, Oliveira AS, Chaves TC, Semprini M, Siessere S, Hallak JE (2007). Use of the Fonseca's questionnaire to assess the prevalence and severity of temporomandibular disorders in Brazilian dental undergraduates. Braz Dent J.

[19] Diracoglu D, Guclu B, Alptekin K, Karan A, Aksoy C (2008). Maximal bite force measurement by the "Istanbul bite force recorder". FTR Bil Der J PMR Sci.

[20] Helenius LM, Hallikainen D, Helenius I, Meurman JH, Kononen M, Leirisalo-Repo M, Lindqvist C (2005). Clinical and radiographic findings of the temporomandibular joint in patients with various rheumatic diseases. A case-control study. Oral Surg Oral Med Oral Pathol Oral Radiol Endod.

[21] Larheim TA, Westesson PL, Sano T (2001). MR grading of temporomandibular joint fluid: association with disk displacement categories, condyle marrow abnormalities and pain. Int J Oral Maxillofac Surg.

[22] Aliko A, Ciancaglini R, Alushi A, Tafaj A, Ruci D (2011). Temporomandibular joint involvement in rheumatoid arthritis, systemic lupus erythematosus and systemic sclerosis. Int J Oral Maxillofac Surg.

[23] Bessa-Nogueira RV, Vasconcelos BC, Duarte AP, Goes PS, Bezerra TP (2008). Targeted assessment of the temporomandibular joint in patients with rheumatoid arthritis. J Oral Maxillofac Surg.

[24] Larheim TA, Storhaug K, Tveito L (1983). Temporomandibular joint involvement and dental occlusion in a group of adults with rheumatoid arthritis. Acta Odontol Scand.

[25] Moen K, Bertelsen LT, Hellem S, Jonsson R, Brun JG (2005). Salivary gland and temporomandibular joint involvement in rheumatoid arthritis: relation to disease activity. Oral Dis.

[26] Ozcan I, Ozcan KM, Keskin D, Bahar S, Boyacigil S, Dere H (2008). Temporomandibular joint involvement in rheumatoid arthritis: correlation of clinical, laboratory and magnetic resonance imaging findings. B-ENT.

[27] Yilmaz HH, Yildirim D, Ugan Y, Tunc SE, Yesildag A, Orhan H, Akdag C (2012). Clinical and magnetic resonance imaging findings of the temporomandibular joint and masticatory muscles in patients with rheumatoid arthritis. Rheumatol Int.

[28] Syrjanen SM (1985). The temporomandibular joint in rheumatoid arthritis. Acta Radiol Diagn (Stockh).

[29] Marbach JJ (1979). Arthritis of the temporomandibular joints. Am Fam Physician.

[30] Tegelberg A, Kopp S (1987). Subjective symptoms from the stomatognathic system in individuals with rheumatoid arthritis and osteoarthrosis. Swed Dent J.

[31] Shovman O, Gilburd B, Zandman-Goddard G, Sherer Y, Orbach H, Gerli R, Shoenfeld Y (2005). The diagnostic utility of anti-cyclic citrullinated peptide antibodies, matrix metalloproteinase-3, rheumatoid factor, erythrocyte sedimentation rate, and C-reactive protein in patients with erosive and non-erosive rheumatoid arthritis. Clin Dev Immunol.

[32] Nell VP, Machold KP, Eberl G, Stamm TA, Uffmann M, Smolen JS (2004). Benefit of very early referral and very early therapy with disease-modifying anti-rheumatic drugs in patients with early rheumatoid arthritis. Rheumatology (Oxford).

[33] Plant MJ, Williams AL, O'Sullivan MM, Lewis PA, Coles EC, Jessop JD (2000). Relationship between time-integrated C-reactive protein levels and radiologic progression in patients with rheumatoid arthritis. Arthritis Rheum.

[34] Gynther GW, Tronje G, Holmlund AB (1996). Radiographic changes in the temporomandibular joint in patients with generalized osteoarthritis and rheumatoid arthritis. Oral Surg Oral Med Oral Pathol Oral Radiol Endod.

[35] Broussard JS, Jr (2005). Derangement, osteoarthritis, and rheumatoid arthritis of the temporomandibular joint: implications, diagnosis, and management. Dent Clin North Am.

